# Epilepsy Associated Gene, *Pcdh7*, Is Dispensable for Brain Development in Mice

**DOI:** 10.3390/genes16080985

**Published:** 2025-08-21

**Authors:** Jennifer Rakotomamonjy, Devin Davies, Xavier Valencia, Olivia Son, Ximena Gomez-Maqueo, Alicia Guemez-Gamboa

**Affiliations:** Department of Neuroscience, Feinberg School of Medicine, Northwestern University, Chicago, IL 60614, USA; jennifer.rakotomamonjy@northwestern.edu (J.R.); ximena.gomezmaqueo@northwestern.edu (X.G.-M.)

**Keywords:** epilepsy, pcdh7, protocadherin, neurodevelopment

## Abstract

**Background/Objectives**: Protocadherin 7 (*Pcdh7*) belongs to the protocadherin family, the largest subgroup of cell adhesion molecules. Members of this family are highly expressed in the brain, where they serve fundamental roles in many neurodevelopmental processes, including axon guidance, dendrite self-avoidance, and synaptic formation. *PCDH7* has been strongly associated with epilepsy in multiple genome-wide association studies (GWAS), as well as with schizophrenia, PTSD, and childhood aggression. Despite these associations, the specific contributions of PCDH7 to epileptogenesis and brain development remain largely unexplored. Most of the existing literature on PCDH7 focuses on its function during cancer progression, with only one study suggesting that PCDH7 regulates dendritic spine morphology and synaptic function via interaction with GluN1. **Methods**: Here, we generate, validate, and characterize a murine null *Pcdh7* allele in which a large deletion was introduced by CRISPR. **Results**: Analysis of embryonic, postnatal, and adult brain datasets confirmed PCDH7 widespread expression. *Pcdh7^+/−^* and *Pcdh7^−/−^* mice present no gross morphological defects and normal cortical layer formation. However, a seizure susceptibility assay revealed increased latencies in *Pcdh7^+/−^* mice, but not in *Pcdh7^+/+^* and *Pcdh7^−/−^* mice, potentially explaining the association of PCDH7 with epilepsy. **Conclusions**: This initial characterization of *Pcdh7* null mice suggests that, despite its widespread expression in the CNS and involvement in human epilepsy, PCDH7 is not essential for murine brain development and thus is not a suitable animal model for understanding PCDH7 disruption in humans. However, further detailed analysis of this mouse model may reveal circuit or synaptic abnormalities in *Pcdh7* null brains.

## 1. Introduction

Protocadherins (PCDH) are the largest subgroup within the cadherin superfamily and function as cell-recognition molecules critical for establishing and maintaining neural circuits [1,2]. The PCDH family is divided into two groups: clustered PCDHs (PCDHα, β, and γ) and non-clustered PCDHs [3]. Non-clustered PCDHs have been implicated in neurodevelopmental processes and disorders when their function is compromised [1,2,4]. Specifically, PCDH7, -10, -12, -17, -18, and -20 mediate axon growth and extension [5,6,7,8,9,10,11,12], and PCDH7, -8, and -10 are implicated in dendritic spine and synapse density [13,14,15,16]. Moreover, PCDH12 and -19 are involved in neural proliferation and migration [17,18], while PCDH19 and -20 are involved in neuronal sorting/positioning [6,17,19]. Pathogenic variants in non-clustered PCDHs have been linked to a wide range of neurodevelopmental and psychiatric disorders. For example, PCDH19-related epilepsy is caused by heterozygous loss-of-function mutations in the X-linked gene, *PCDH19* [20,21]. *PCDH12* variants have been associated with schizophrenia [22], and bi-allelic pathogenic variants lead to seizures, microcephaly, and white matter abnormalities [9,23,24,25,26]. *PCDH8*, -9, and -10 de novo variants have been linked to autism [16,27,28], while variants in *PCDH17* confer a risk for mood disorders [29].

Although much of the research on PCDH7 pathogenic variants has primarily focused on its role in cancer [30,31], genome-wide association studies (*GWAS*) have linked *PCDH7* to various neurological disorders, including epilepsy [32,33], schizophrenia [34], PTSD [34], and childhood aggression [35]. PCDH7 is downregulated by MeCP2 and upregulated in the brains of Mecp2 KO mice [36], suggesting a connection between PCDH7 and the neurodevelopmental disorder Rett syndrome. Furthermore, recent findings suggest that haploinsufficiency of PCDH7 might contribute to the mental developmental delay observed in patients with proximal 4p deletion syndrome [37]. Additionally, PCDH7 is located in the excitatory synaptic cleft [38] and interacts with the NMDA receptor subunit GluN1 [14]. These insights collectively highlight a possible role for PCDH7 in both brain development and synaptic function.

Pcdh7 exhibits a region-specific expression pattern within the murine developing cerebral cortex [38]; however, the consequences of pcdh7 loss during brain development have yet to be investigated. The only report of a Pcdh7 null mouse focused on bone homeostasis [14], omitting any examination of brain morphology. Most research investigating the role of *pcdh7* during neurodevelopment has been performed in Xenopus or zebrafish using morpholinos. These studies have shown that pcdh7 regulates the histogenesis of the embryonic ectoderm [1], neural tube formation [39], and axon growth [40] in Xenopus morphants. Morpholino knockdown in zebrafish also results in impaired neural stem cell differentiation and disrupted neural architecture during embryonic brain development. In contrast, *pcdh7* null zebrafish embryos did not exhibit any phenotypic defects [41]. While these findings support a role for *pcdh7* during brain development, the commonly observed discrepancies between the mutant and morphant phenotypes [11,42] complicate the interpretation of these results.

Here, we generated, validated, and characterized a murine *Pcdh7* null allele containing a large CRISPR-mediated deletion. Analysis of embryonic, postnatal, and adult brain datasets confirmed PCDH7 widespread expression. Examination of brain morphology and cortical layer formation showed no abnormalities in both *Pcdh7^+/−^* and *Pcdh7^−/−^* mice. However, while *Pcdh7^+/−^* mice exhibited increased latencies to seizures, this was not observed in *Pcdh7^−/−^* nor *Pcdh7^+/+^* mice, potentially explaining the association of PCDH7 with epilepsy. This initial characterization of the *Pcdh7* mouse model reveals that, despite widespread expression in the CNS and its association with human epilepsy, Pcdh7 function is not essential for murine brain development.

## 2. Materials and Methods

### 2.1. Mice

All experiments were approved by the Northwestern University Animal Care and Use Committee in accordance with the NIH Guide for the Care and Use of Laboratory Animals and the Animal Welfare Act. Animals were housed on a 14-h light/10-h dark schedule, with food and water available *ad libitum*. C57BL/6J mice were used in all experiments.

### 2.2. CRISPR Gene Editing

Generation of Pcdh7 null mice was carried out by using the CRISPR/Cas9 system. Briefly, we selected four gRNAs using the CRISPOR program [43] based on specificity scores, location, and predicted cleavage efficiency to minimize off-target effects. The four gRNAs were synthetized using the IDT AltR system, and the high-fidelity Cas9 protein was used. All four gRNAs in conjunction with the Cas9 protein were electroporated into fertilized murine eggs (zygotes). The injected embryos were then transferred into a pseudo-pregnant female mouse. Once pups were born, screening for gene editing events was performed via PCR amplification of the *Pcdh7* exon 1 locus followed by Sanger sequencing. The full *Pcdh7* exon1 locus was amplified using *Pcdh7* primer 2F: 5′-TCCCCTCCCTCTCGTTTCTT-3′ and *Pcdh7* primer 1R: 5′-ACACTACCTTTCCCTTAGATTCCA-3′. *Pcdh7* WT amplicon = 3560 bp. PCR products were separated on a 1% agarose gel. Smaller amplicons suggested deletions; thus, the bands were excised and gel-purified using the Qiagen gel purification kit (Qiagen, Germantown, MD, USA). Purified PCR products were submitted for Sanger sequencing. After sequencing, two surviving founders were identified. Male 9659 (which carried 2 mutant alleles, including the 1145 bp deletion), and Male 9660, which also carried 2 mutant alleles, including a large 2243 bp deletion. Multiple alleles in a single founder are commonly found when mutations are generated directly in the embryo, and this is due to CRIPSR-mediated editing in the zygote after the first cellular division. Because of the complexity of the mutations, founders were bred with WT females to segregate the alleles. F1 mice carrying the KO allele were recovered with a 50% transmission of the KO alleles (1145 bp deletion for KO1 and the 2243 bp deletion for KO2).

### 2.3. Genotyping

Genotyping was performed by PCR amplification using the following primers: Forward: 5′-TCCCCTCCCTCTCGTTTCTT-3′; Reverse-1 (KO): 5′-ACGTCTCCCACTACGGTACA-3′; Reverse-2 (WT): 5′-CCCTCCCACAATGCTGTAGT-3′. PCR products were separated on a 1% agarose gel. *Pcdh7* WT and KO allele products are 273 bp and 382 bp, respectively.

### 2.4. RNA Isolation and RT-PCR

RNA isolation was performed using Direct-zol^TM^ RNA miniprep plus isolation kit (Zymo Research, Irvine, CA, USA), and RNA concentration was measured using a Nanodrop. cDNA was synthesized from 5 µg RNA using the Superscript III reverse transcriptase (Thermo Fisher Scientific, Waltham, MA, USA) cDNA synthesis kit. RT-PCR was performed according to the manufacturer’s instructions. PCR amplification using the following primers: Exon 1: Forward: 5′-TCCCCTCCCTCTCGTTTCTT-3′; Reverse: 5′-ACGTCTCCCACTACGGTACA-3′; Exon 2–3: Forward: 5′-TCCCCTCCCTCTCGTTTCTT-3′; Reverse: 5′-CCCTCCCACAATGCTGTAGT-3′. PCR products were separated on a 1% agarose gel. Exons 1 and 2–3 products are 173 bp and 267 bp, respectively.

### 2.5. Histology and Immunohistochemistry

Brains were post-fixed in PFA 4% overnight, cryoprotected in 30% sucrose until sunk, and snap frozen embedded in OCT. 25 µm coronal sections were obtained using a cryostat (Leica CM1860, Leica Biosystems, Deer Park, IL, USA). For histological measurements, both cortical and CA1 hippocampal thicknesses were assessed across multiple serial sections and averaged to obtain a representative thickness for each individual mouse brain. For immunohistochemistry, slides were incubated overnight at 4 °C with the primary antibody, then with a fluorescently labeled secondary antibody (Invitrogen, Waltham, MA, USA) for 2 h at room temperature (RT), and counterstained with Hoechst 33342 (2 µg/mL, Thermo Fisher Scientific, Waltham, MA, USA). Fluorescent signal was detected using an inverted microscope (BZ-X710 Fluorescence Microscope, Keyence, Itasca, IL, USA). Primary antibodies include anti-Satb2 (1:200, Abcam, Waltham, MA, USA) and anti-NeuN (1:500, Thermo Fisher Scientific, Waltham, MA, USA).

### 2.6. Flurothyl Seizure Induction

Flurothyl seizure induction was performed as previously described [44,45]. Briefly, the mice were individually placed in a sealed induction chamber. Flurothyl (2,2,2-trifluroethyl ether, Sigma-Aldrich, St. Louis, MO, USA) was introduced by a syringe pump (Genie Touch, Kent Scientific, Torrington, CT, USA) at a constant rate of 20 μL/min. Following flurothyl induction, reviewers blinded to the genotype recorded the latency to the first myoclonic (MJ) and generalized tonic-clonic seizure (GTCS). At the onset of GTCS, the mouse was immediately placed in a holding cage for recovery. All mice were euthanized at the end of the experimental session.

### 2.7. Western Blot Analysis

Immediately following the flurothyl protocol, brain tissue was weighed and lysed in 1X RIPA buffer (Cell Signaling Technology, Danvers, MA, USA), supplemented with protease inhibitor cocktail (Thermo Fisher Scientific, Waltham, MA, USA) at a 1:50 ratio (weight: volume). Protein concentration was determined by BCA assay (Thermo Fisher Scientific, Waltham, MA, USA). Samples were denatured in Laemmli buffer (Bio-Rad, Hercules, CA, USA) by boiling for 5 min at 95 °C. Proteins (30 µg) were resolved on 10% acrylamide gels by SDS-PAGE and transferred onto PVDF membranes. Membranes were blocked in Intercept^®^ (TBS) blocking buffer (LICORbio, Lincoln, NE, USA), and primary antibodies were incubated in the same buffer supplemented with 0.2% Tween20 at 4 °C overnight. Primary antibodies were anti-GFAP (1:5000, Invitrogen, Waltham, MA, USA) and anti-α-Tubulin (1:10,000, Abcam, Waltham, MA, USA). Infrared (IR) secondary antibodies were incubated in Intercept^®^ (TBS) blocking buffer (LICORbio, Lincoln, NE, USA) supplemented with 0.2% Tween20 and 0.01% SDS at a 1:10,000 dilution for 1 h at RT. IR fluorescent blots were imaged with the Odyssey^®^ Fc imaging system (software version 1.0.37, LICORbio, Lincoln, NE, USA). Image acquisition and quantification were performed with Image Studio (version 5.2.5, LICORbio, Lincoln, NE, USA).

### 2.8. Statistical Analysis

Statistical analyses were performed using GraphPad Prism 10 (version 10.4.0, GraphPad Software, Boston, MA, USA). Results are presented as mean ± standard deviation, unless mentioned otherwise. One-way analysis of variance (ANOVA) followed by Tukey’s post hoc multiple comparisons test was used after verification that normality and homoscedasticity criteria were met. A *p*-value < 0.05 was considered statistically significant.

## 3. Results

### 3.1. Pcdh7 Is Widely Expressed in Mice and Human Brain During Development

**Figure 1:**
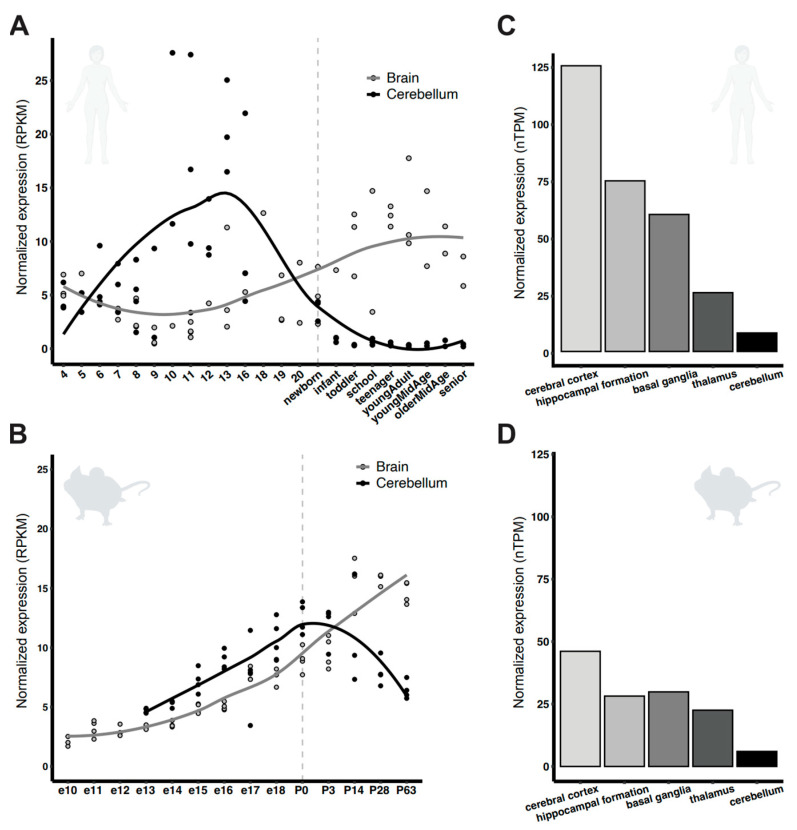
*PCDH7* expression in human and murine brain. Normalized expression across developmental stages in (**A**) human, and (**B**) mouse. The vertical line separates prenatal and postnatal stages. Data was adapted from [46]. Normalized expression across the adult brain regions in (**C**) human and (**D**) mouse. Data was adapted from [47].

Most studies on PCDH7 have focused on its role in cancer, despite reports that, like most PCDHs, it is highly expressed in the nervous system [1,14]. Thus, we first conducted a comprehensive analysis of both murine and human embryonic, postnatal, and adult brain datasets to better understand *PCDH7* expression patterns [46,47]. We found that *PCDH7* is expressed throughout brain development, with higher expression after birth in both humans and mice (Figure 1A,B). This data is in line with previous studies reporting *Pcdh7* expression in the rodent developing brain [1,41,48]. In contrast, *PCDH7* expression is higher in prenatal timepoints, decreasing after birth in the cerebellum of both humans and mice (Figure 1A,B). *PCDH7* is expressed in multiple regions of both the mouse and human central nervous system (CNS), with the highest expression observed in the cortex, followed by the hippocampus, basal ganglia, and thalamus, with the least expression in the cerebellum (Figure 1C,D). Finally, relative *PCDH7* expression is higher in the human brain than in the mouse brain (Figure 1C,D).

### 3.2. Generation and Validation of a Pcdh7 Null Mouse

**Figure 2:**
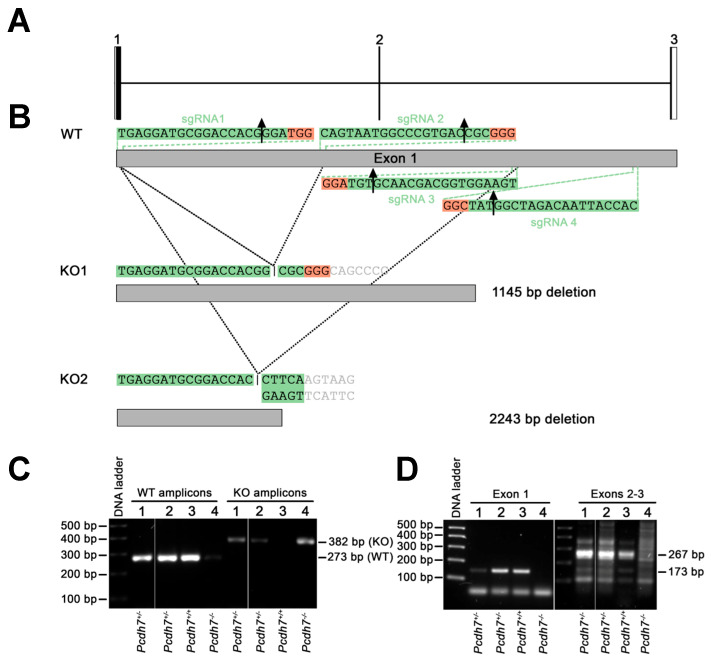
Generation of the Pcdh7 null mouse model. Schematic representation of the WT mouse *Pchd7* genomic locus (**A**) and the sgRNA target sites for the generation of two Pcdh7 null mouse strains (**B**). Conventional PCR analysis of genomic DNA from the 1145-bp-deletion Pcdh7 mice, *Pcdh7^+/+^* WT band, *Pcdh7^+/−^* WT and KO bands, *Pcdh7^−/−^* KO band (**C**). Confirmation of Pcdh7 loss of expression by RT-PCR amplifying a 173-bp cDNA fragment in exon 1 and a 267-bp fragment spanning exons 2 and 3 (**D**).

The Pcdh7 gene contains four exons, with exon 1 encoding most of the protein, including the extracellular and transmembrane domains (Figure 2A). Because Pcdh7 has several potential ATG start sites, a complete disruption of exon 1 is necessary for the generation of a Pcdh7 null mouse model. Thus, we targeted 4 regions of exon 1 with multiple gRNAs to obtain a CRISPR cleavage disruption. We selected four gRNAs using the CRISPOR program [43] based on specificity scores, location, and predicted cleavage efficiency. Their position in exon 1 and the resulting deletions are depicted in Figure 2B. The four gRNAs were electroporated into the murine zygotes to disrupt Pcdh7 exon 1. IDT AltR system gRNAs and the high-fidelity Cas9 protein were electroporated into the zygotes to ensure that the CRISPR complex was transient and reduce possible off-target effects. Screening for gene editing events was performed via PCR amplification of the Pcdh7 exon 1 locus followed by Sanger sequencing. PCR protocols and primer sets were optimized to amplify the full Pcdh7 exon 1 locus. Two founder male mice were then identified: KO1, harboring the 1145 bp deletion, and KO2, with the 2243 bp deletion. The disruption of the Pcdh7 gene was validated by the PCR using genomic DNA (Figure 2C), and the reduction of Pcdh7 expression was confirmed by RT-PCR using cDNA (Figure 2D).

Founders were set up in breeding trios with WT females. F1 mice carrying the KO allele were identified after successful pairing. KO1 F1 consisted of two litters, 20 mice (8 males, 12 females), with a 50% transmission of the 1145 bp deletion as expected. KO2 F1 consisted of three litters for a total of 25 mice (14 males, 11 females) with 12 of 25 mice carrying the 2243 bp deletion. F1 and subsequent *Pcdh7^+/−^* breeding pairs generated *Pcdh7^+/+^*, *Pcdh7^+/−^*, and *Pcdh7^−/−^* mice at the expected frequencies (Table 1). Adult mice from all genotypes presented with similar body sizes and no overt health issues.

### 3.3. Pcdh7^+/−^ but Not Pcdh7^−/−^ Mice Present with Increased Seizure Latencies

**Figure 3:**
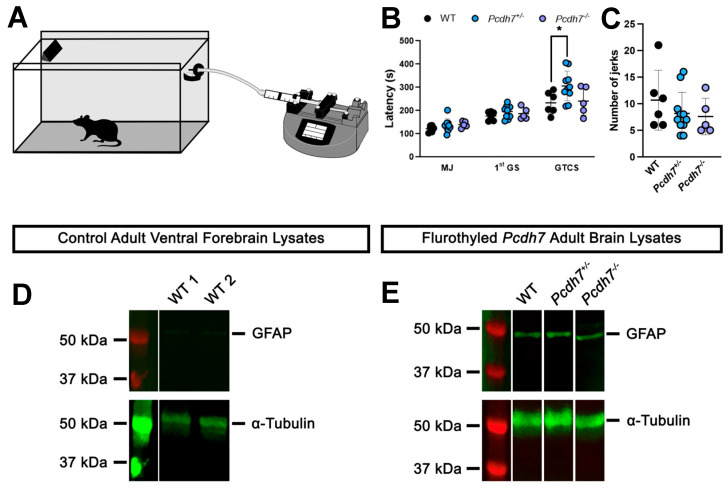
Seizure susceptibility in the Pcdh7 null mouse model. Schematic representation of the experimental setup of the flurothyl-induced seizure assay (**A**). Latencies to first myoclonic jerk (MJ), first generalized seizure (GS), characterized by transient loss of balance, and general tonic-clonic seizure (GTCS), characterized by loss of balance, wild running, and hopping behavior, were recorded (**B**), as well as the number of myoclonic jerks exhibited before the occurrence of the first GS (**C**). Western blot analyses of GFAP and α-Tubulin expression in control (**D**) or flurothyled (**E**) brain lysates. Data are shown as mean ± SD. n = 5–10 mice/genotype. * *p* = 0.0200, One-way Anova followed by Tukey’s multiple comparison test.

PCDH7 has been strongly linked to epilepsy [32]; however, we did not observe spontaneous seizures in any mice from all genotypes at any developmental time point. Next, we induced generalized seizures using the GABA_A_ receptor function inhibitor flurothyl (Figure 3A). Mice exposed to flurothyl express a series of characteristic seizure behaviors, beginning with brief myoclonic jerks (MJ), which progress to generalized seizures (GS) and eventually culminate in generalized tonic-clonic seizures (GTCS). The average latencies to MJ and the first GS were comparable among genotypes (122 ± 14 s, 137 ± 28 s, and 140 ± 14 s to first MJ in WT, Pcdh7^+/−^, and Pcdh7^−/−^ mice, respectively; 177 ± 19 s, 196 ± 27 s, and 187 ± 25 s to first GS MJ in WT, Pcdh7^+/−^, and Pcdh7^−/−^ mice, respectively). However, the average latency to GTCS was significantly increased in heterozygous mice (306 ± 64 s) when compared with their WT littermates (234 ± 48 s) (Figure 3B), and no differences were observed in the number of jerks regardless of genotype (Figure 3C). Finally, flurothyl induced astrocyte activation, as observed by GFAP expression in Western blots of brain lysates from all genotypes. 

### 3.4. Pcdh7^+/−^ and Pcdh7^−/−^ Mice Exhibit No Gross Brain Abnormalities

**Figure 4:**
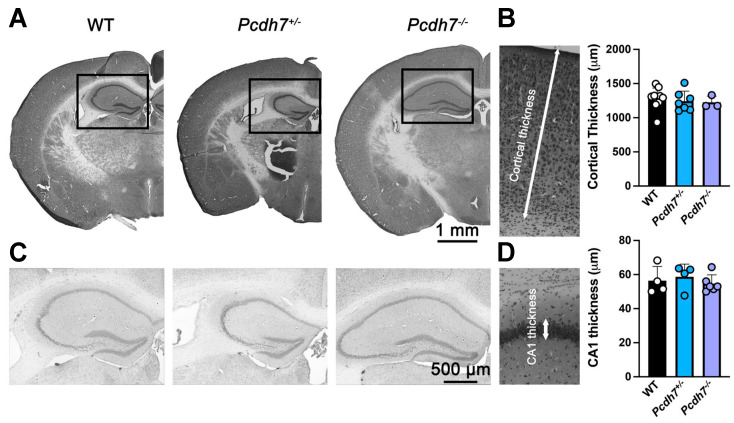
Histological analysis of the Pcdh7 mouse model. Cresyl violet staining was performed on coronal sections of adult Pcdh7 mice (**A**). The graph represents the thickness measurement of the lateral cortex (**B**). Hippocampal formation from insets shown in (**A**,**C**). The graph represents the thickness measurement of the hippocampus CA1 regions (**D**). No significant differences were observed between genotypes. Data are shown as mean + SD. Each data point represents a measurement in one coronal section. n = 3 mice/genotype.

**Figure 5:**
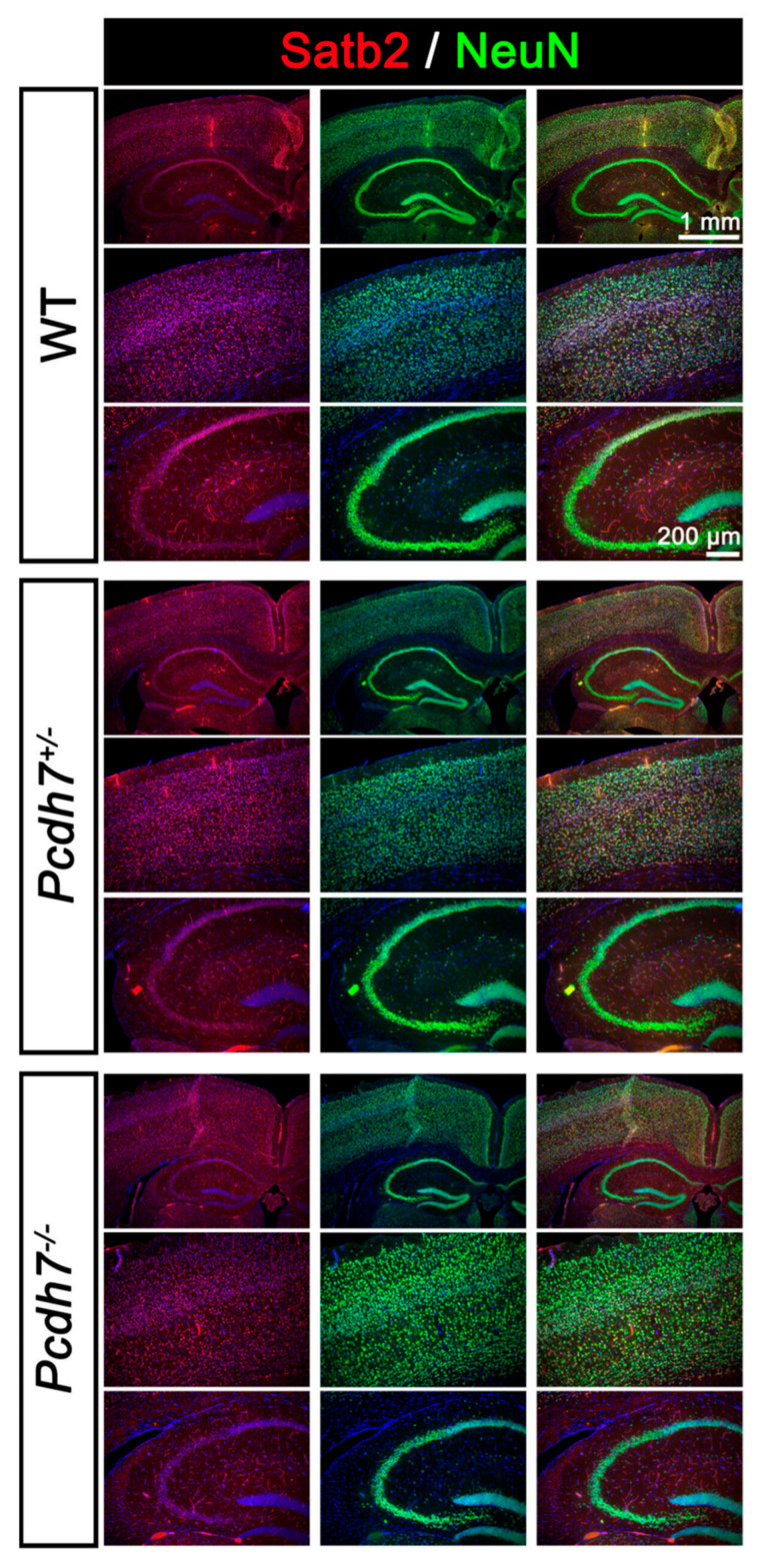
Immunostaining of coronal sections of adult *Pcdh7* mice. Hippocampal formation is shown in the insets. Satb2 (red), NeuN (green), and DAPI (blue). No differences were observed between genotypes.

Morpholino knockdown of *pcdh7* in Xenopus and zebrafish embryos results in CNS defects. We therefore examined gross brain morphology in our newly generated *Pcdh7* mouse model. Histological analysis of WT, *Pcdh7^+/−^*, and *Pcdh7^−/−^* brains showed no gross morphological abnormalities. As *Pcdh7* is highly expressed in the cerebral cortex of both humans and mice (Figure 1), we measured the cortical and hippocampus CA1 thickness (Figure 4). We also analyzed cortical layer formation using immunostaining with the pan-neuronal marker NeuN and the pyramidal neuron marker Satb2 (Figure 5). NeuN is found in the nucleus of mature neurons, and it was used to visualize neuronal density and potential neuronal loss due to the lack of Pcdh7. Satb2 is required during cortical development for the cell fate specification of callosal projection neurons, and it is expressed in excitatory neurons in the adult. No differences between *Pcdh7^+/−^* and *Pcdh7^−/−^* brains and *Pcdh7*^+/+^ controls were observed (Figure 4 and Figure 5), suggesting that loss of *Pcdh7* in mice does not overtly alter brain structure.

## 4. Discussion

Pathogenic variants in protocadherin genes have been implicated in several neurological diseases. Specifically, PCDH7 has been associated with multiple neurological disorders, including epilepsy [32,34,35,36,37,49]. *Pcdh7* is predominantly expressed in the developing murine brain, in both excitatory and inhibitory neurons, with an enrichment in synapses [38]. Our data analysis also confirms differential PCDH7 expression throughout development, with higher levels after birth in both human and mouse nervous systems, supporting the role of PCDH7 in synapse formation and function. We observed robust *Pcdh7* expression, particularly in brain regions implicated in epilepsy, such as the hippocampus and cortex, consistent with previous studies in rodents [1,41,48,50]. Animal models lacking PCDHs have revealed important functions in various aspects of neurodevelopment, including axon outgrowth [5,12], dendrite branching [51,52], dendritic spine density [15], and synaptic formation and function [53]. Thus, to start exploring the impact of loss of pcdh7 during brain development, in general, and in epilepsy, in particular, we generated a *Pcdh7* null mouse model.

Previous studies have reported that disruption of pcdh7 causes neural defects during brain development in zebrafish embryos [41]. However, these defects are only observed using morpholino knockdown and were not present in zebrafish with a genetic mutation of *pcdh7* [41]. These phenotypic differences may reflect a lack of genetic compensation in response to acute morpholino knockdown, as previously reported [54,55]. Nevertheless, the lack of brain abnormalities in our newly generated *Pcdh7^+/+^*, *Pcdh7^+/−^*, and *Pcdh7^−/−^* mice is consistent with *pcdh7^−/−^* zebrafish embryos and with other protocadherin null mouse models of disease. Of particular interest are the *Pcdh19* null mice, which have been reported to be healthy, fertile, and with no gross brain defects [56]. In contrast, *PCDH19* patients develop infantile seizures with variable cognitive defects and cortical dysplasia [17,21]. Another example is the *Pcdh12* null mice, also reported to be viable and show no morphological abnormalities [57], while the patients carrying bi-allelic pathogenic variants present with seizures, microcephaly, and white matter abnormalities [9,23,24,25,26]. The absence of gross brain defects in our *Pcdh7* null mouse model may present functional redundancy of *Pcdh7* that was within expectations.

The association of *PCDH7* with genetic generalized epilepsy [32,33] prompted us to investigate whether our *Pcdh7* mouse model exhibits seizures. While we did not observe spontaneous seizures in any genotype, *Pcdh7^+/−^* mice, but not *Pcdh7^+/+^* and *Pcdh7^−/−^* mice, presented with increased latency to seizures induced by flurothyl. The *Pcdh7^+/−^* genotype is the one carried by patients identified with epilepsy through GWAS. Although the mechanisms by which PCDH variants lead to neurological disorders are not fully understood, insights from the well-studied PCDH19 suggest that its unique X-linked inheritance pattern may disrupt cellular adhesion affinities, an occurrence potentially exacerbated by X-inactivation [17]. Unlike *PCDH19*, which is located on the X chromosome, *PCDH7* and other members of the PCDH family reside on autosomes. Nonetheless, evidence indicates that these proteins can undergo random monoallelic expression [58]. This may cause symptoms in individuals with heterozygous variants due to an imbalance in PCDH expression, potentially leading to similar disrupted adhesion affinities to those seen in PCDH19-related epilepsy. In cases where all cells either express only the WT PCDH or none at all, no symptoms occur. This phenomenon is exemplified by hemizygous males with *PCDH19* mutations, who typically do not exhibit symptoms associated with PCDH19-related epilepsy [21]. Therefore, our findings may provide an explanation for the association of *PCDH7* with epilepsy in humans.

Most of the genes known to cause or contribute to epilepsy can be traced to the synapse, including PCDH7, which is located in the excitatory synaptic cleft [38], and interacts with the NMDA receptor subunit GluN1 [14]. Interestingly, cell-autonomous knockdown of PCDH7 did not affect the NMDA receptor current [14]. Whether the NMDA receptor current is affected by PCDH7 in all components of the circuit, and not only in the postsynaptic cell, remains an open line for future investigation.

In summary, our characterization of *Pcdh7* null mice suggests that, although *Pcdh7* is widely expressed in the CNS, it is not required for murine brain development and thus not a suitable animal model for understanding PCDH7 disruption in humans. However, given the increased latency to seizures in *Pcdh7* heterozygous mice and the identification of multiple roles for *Pcdh7* in neurodevelopmental processes in vitro, further detailed analysis of this mouse model may reveal circuit or synaptic abnormalities in *Pcdh7* null brains.

## Figures and Tables

**Table 1:** Breeding *Pcdh7^+/−^* mice generated litters with the expected genotype and sex frequencies.

		**Females**	**Males**	**Total**	
**Numbers**		33	40	73	
**%**		45	55	100	
		**WT**	**Pcdh7^+/−^**	**Pcdh7^−/−^**	**Total**
**Numbers**		15	38	17	70
**%**		21	54	24	100
		**WT**	**Pcdh7^+/−^**	**Pcdh7^−/−^**	**Total**
**Numbers**	Females	7	15	8	30
	Males	8	23	9	40
**%**	Females	23	50	27	100
	Males	20	58	23	100

## Data Availability

PCDH7 expression profiles were obtained from The Evo-devo mammalian organs project (https://apps.kaessmannlab.org/evodevoapp, accessed on 7 May 2025) and The Human Protein Atlas (https://www.proteinatlas.org, accessed on 7 May 2025).

## Abbreviations

The following abbreviations are used in this manuscript:

PCDH7	Protocadherin 7
CRISPR	Clustered Regularly Interspaced Short Palindromic Repeats
HET	Heterozygous
KO	Knock-out
MJ	Myoclonic jerk
GTCS	Generalized tonic-clonic seizure
GS	Generalized seizure
gRNA	Guide RNA
